# Supplementary material for: XPC deficiency increases risk of hematologic malignancies through mutator phenotype and characteristic mutational signature
Source: Nat Commun. 2020 Nov 17;11:5834. doi: 10.1038/s41467-020-19633-9 (PMC7672101; doi:10.1038/s41467-020-19633-9)
Supplement: Supplementary file 1 — Supplementary Information [file 41467_2020_19633_MOESM1_ESM.pdf]

XPC deficiency increases risk of hematologic malignancies through mutator phenotype and characteristic mutational signature

Yurchenko et al.

Supplementary Figure 1

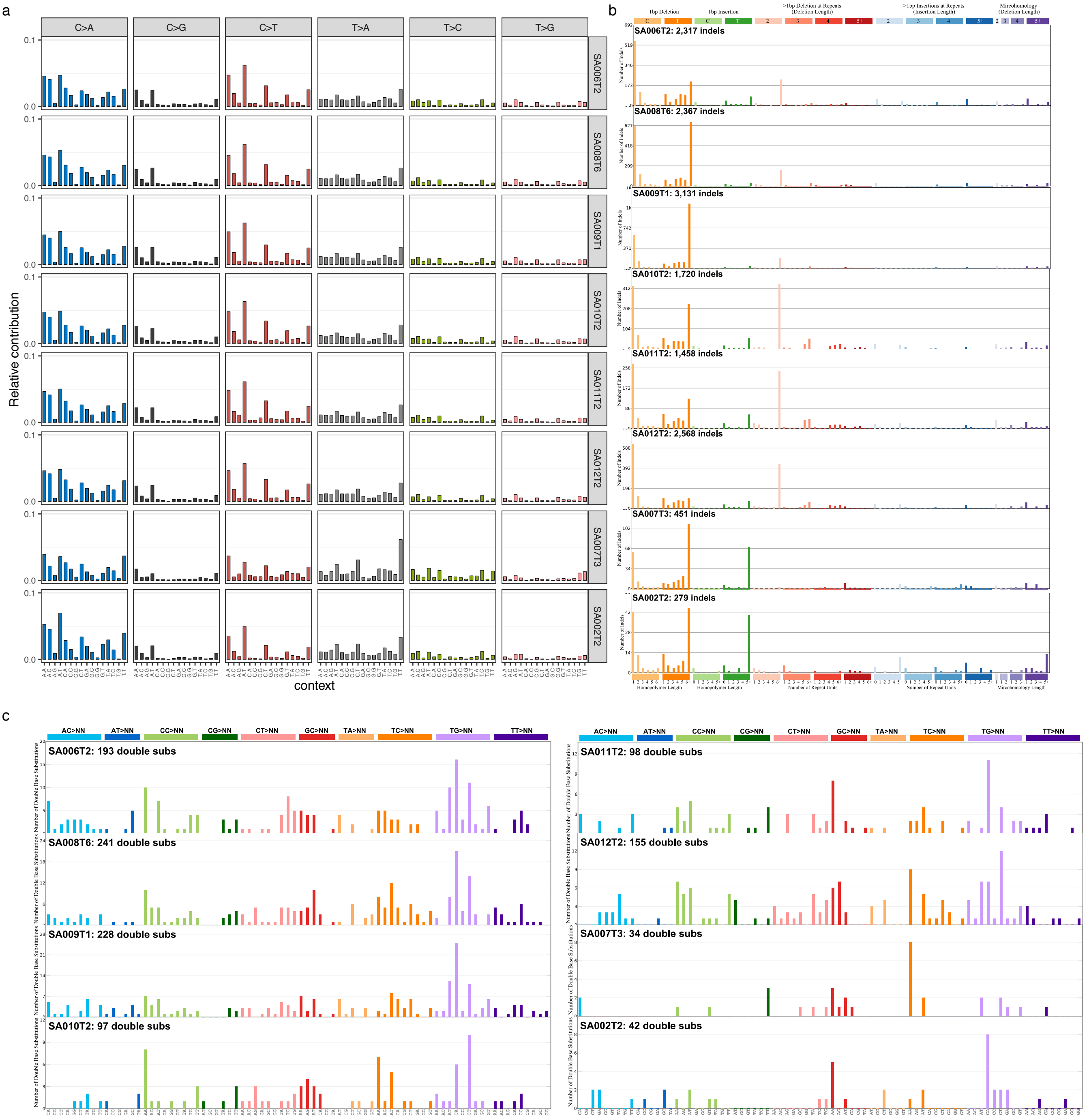

Supplementary Figure 1. Individual mutational profiles of XP-C tumors for different types of mutations.

a, Single base substitutions (SBS).

b, Indels (ID).

c, Double base substitutions (DBS).

Supplementary Figure 2

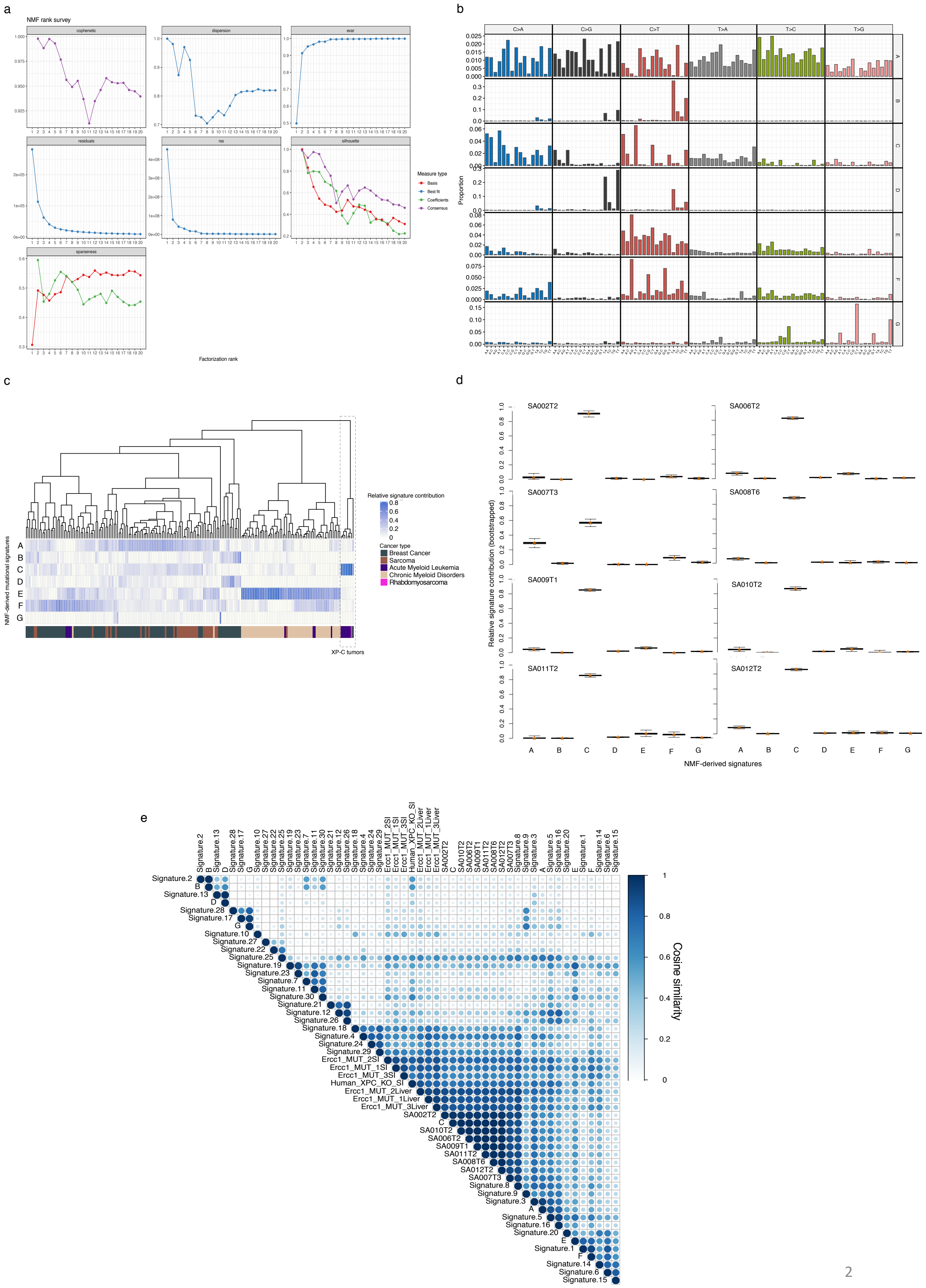

Supplementary Figure 2. Non-negative Matrix Factorization-derived (NMF) mutational profiles and their contribution in XP-C and sporadic cancers.

a, Factorization ranks of NMF and diagnostic plots. The model (K=7) was chosen based on the inflation of RSS (Residual Sum of Squares) and evar (explained variance achieved by a model).

b, Trinucleotide profiles of NMF-derived mutational signatures.

c, Relative contribution of the NMF-derived mutational signatures in XP-C tumors and sporadic tissue-matched samples (unsupervised hierarchical clustering, relative contribution was inferred using quadratic programming-based algorithm (Huang et al., 2018)). XP-C tumors group together, and their mutational profiles are characterized by high level of Signature "C".

d, Bootstrapped estimation (10000 replications, boxes depict the interquartile range (25–75% percentile), lines the median, whiskers - 1.5× the IQR below the first quartile and above the third quartile) of relative contribution of the NMF-derived signatures in XP-C tumors (quadratic programming-based algorithm). Signature "C" dominates in all the samples with very little level of variation and only in breast sarcoma sample (SA007T3) is slightly depleted.

e, Cosine similarity matrix between NMF-derived mutational signatures (A-G), COSMIC mutational signatures (Signatures 1-30), mutational profiles of XP-C tumors (SA00..), and Ercc1 and XPC deficient organoid cultures.

Supplementary Figure 3

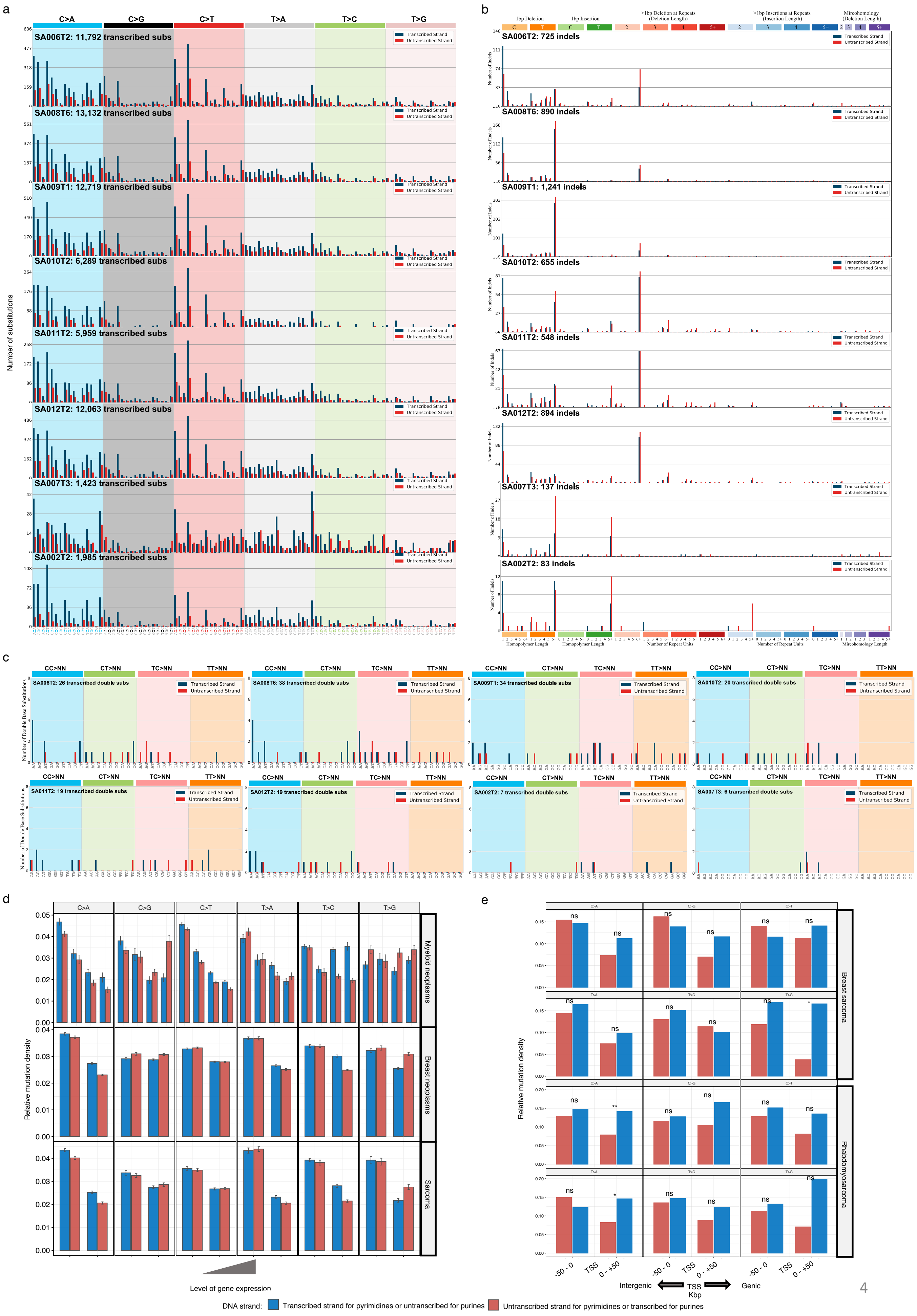

Supplementary Figure 3. Transcriptional bias (TRB) in XP-C tumors and sporadic cancers.

a, Stranded mutational profiles of XP-C tumors in genic regions for single base substitutions (SBS). Canonical notation depicts mutations from pyrimidines (blue – transcribed for mutations from pyrimidines, untranscribed for mutations from purines; red – untranscribed for mutations from pyrimidines, transcribed for mutations from purines).

b, Stranded mutational profiles of XP-C tumors in genic regions for indels (ID).

c, Stranded mutational profiles of XP-C tumors in genic regions for double base substitutions (DBS).

d, TRB does not change significantly with the level of gene expression in sporadic tumors (SEM are indicated; myeloid neoplasms n=65, breast cancer n=91, sarcoma n=34).

e, Relative mutation density for mutations from purines and pyrimidines in genic regions and neighboring intergenic regions of XP-C samples (SA007T3 and SA002T2, breast sarcoma (n=1) and rhabdomyosarcoma (n=1) respectively, Poisson two-sided test, P: ns – nonsignificant, * < 0.5, ** < 0.01, *** < 0.001).

Supplementary Figure 4

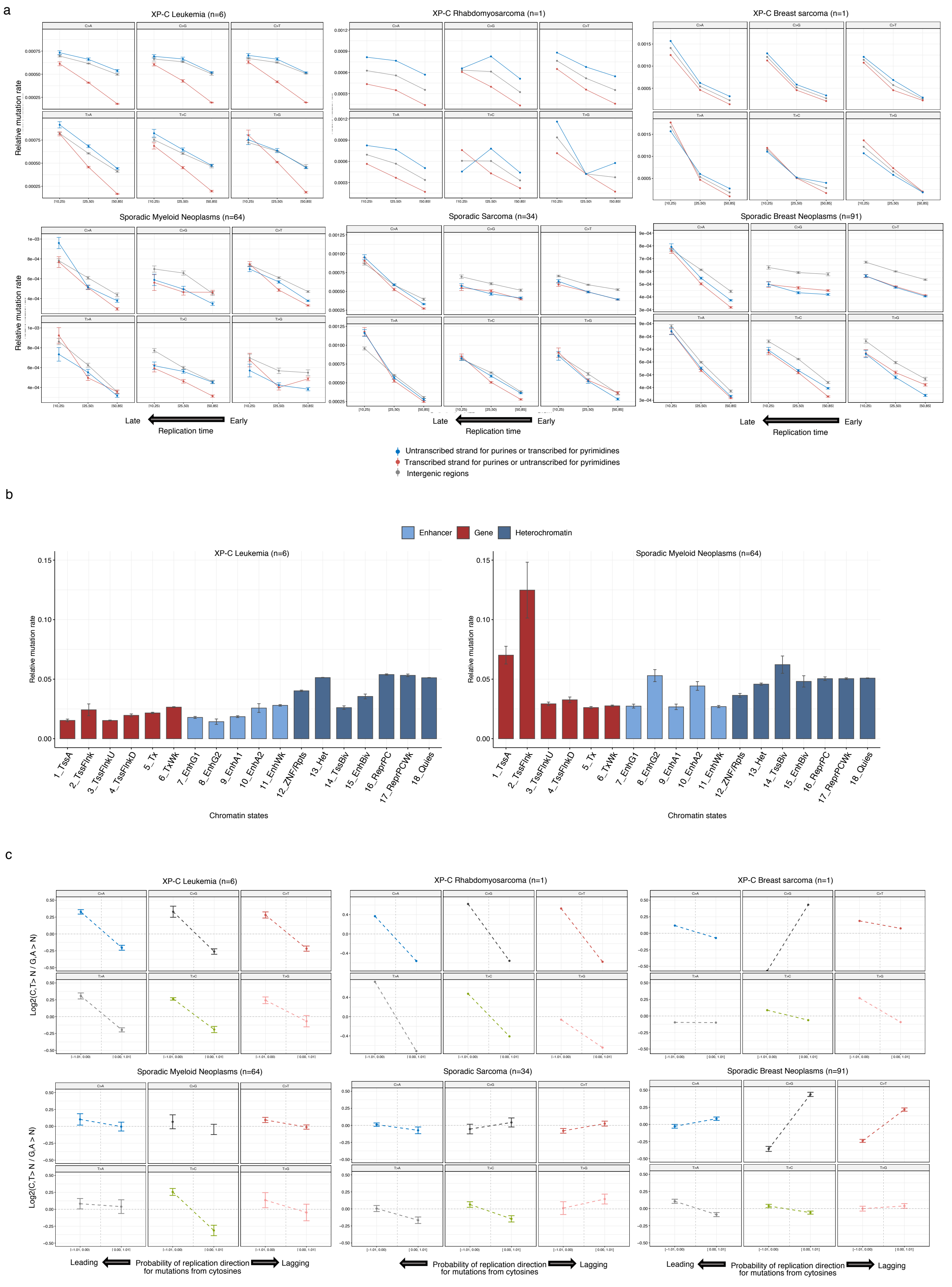

Supplementary Figure 4. Genomic landscape of mutagenesis in XP-C internal tumors (n – number of cancer samples used).

a, Replication timing and intensity of mutagenesis on the transcribed and untranscribed DNA strands as well in intergenic regions of XP-C samples and tissue-matched sporadic cancers (SEMs are indicated). The transcribed strand for pyrimidines (or untranscribed for purines, blue) behaves as intergenic regions genome-wide due to the absence of GG-NER in XP-C tumors (except breast sarcoma, with relatively low amount of Signature "C") while this effect is very weak in sporadic cancers due to the different mutagenesis process and functional GG-NER.

b, Relative mutation rate in different chromatin states (ChromHMM) for XP-C leukemia and sporadic myeloid neoplasms (SEMs are indicated).

c, Replication direction (leading and lagging) and relative intensity of mutagenesis (replication bias) in XP-C and tissue-matched sporadic tumors (SEMs are indicated). Enrichment of the relative mutagenesis on the leading strand from pyrimidines (C and T) corresponds to the enrichment on the lagging strands from purines (G and A). For all six mutational classes we observe strong enrichment of mutations from purines on the lagging DNA strand of XP-C leukemia and rhabdomyosarcoma samples. The effect is evident but less pronounced in XP-C breast sarcoma. In sporadic cancers the effect is weak or work in opposite direction.

Supplementary Figure 5

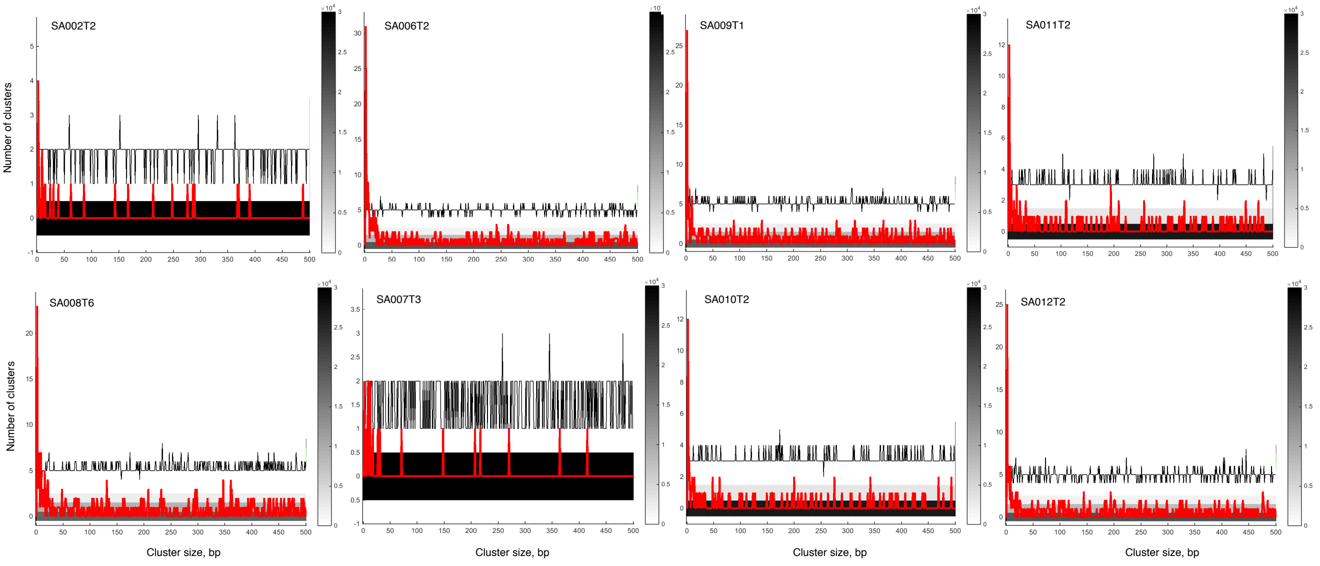

Supplementary Figure 5. The assessment of the length of clustered mutation events for 1-500bp distances.

Observed inter-mutation distance (red) is compared to the density distribution of 30000 simulations (black) with similar number of mutations and trinucleotide contexts. Strong enrichment of clustered events is evident at short cluster distances in the all the samples.

Supplementary Figure 6

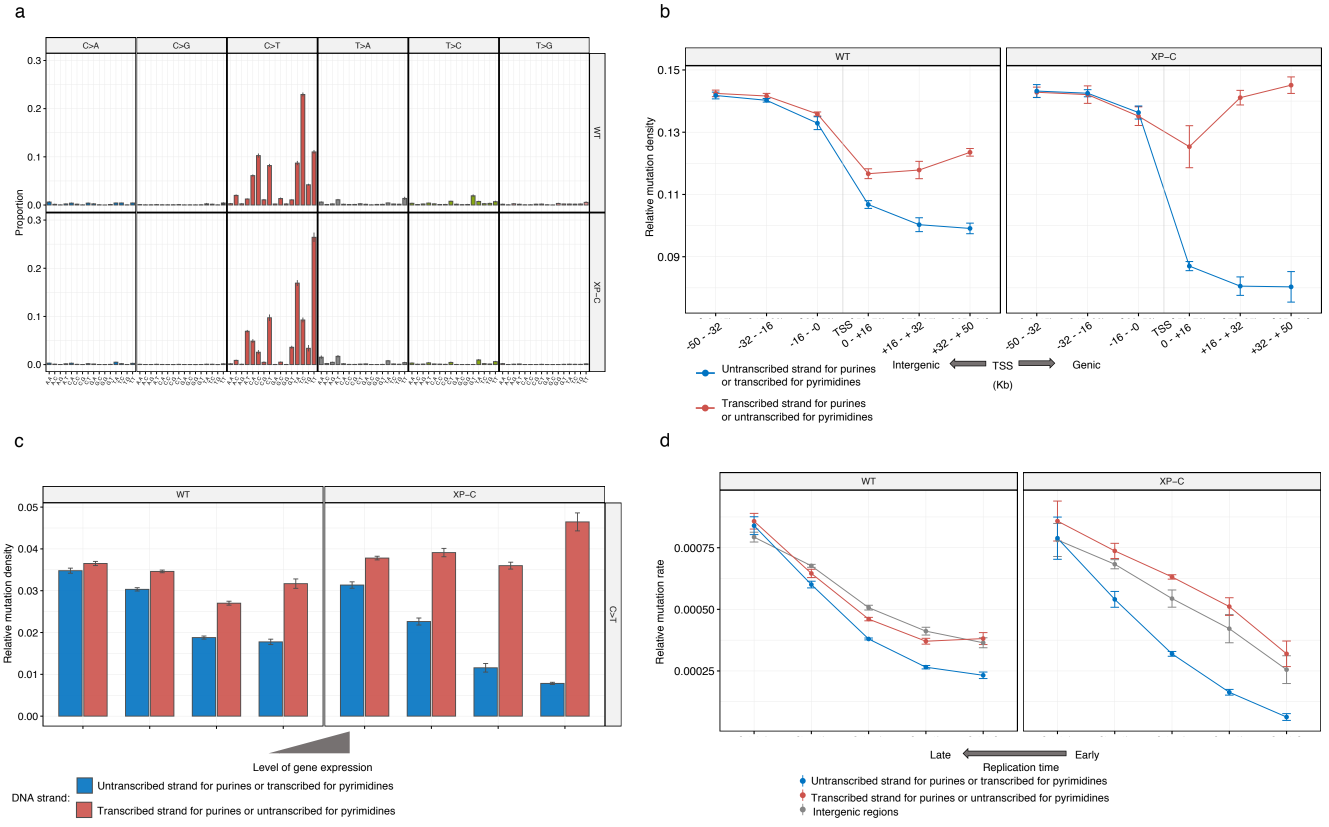

Supplementary Figure 6. Genomic mutational landscape of WT (n=7) and XP-C (n=5) cutaneous squamous cell carcinoma (cSCC) samples.

a, Trinucleotide-context mutational profiles (SEM intervals are shown). X-axis represents the nucleotides upstream and downstream of mutation. The mutational profiles of both WT and XP-C cSCCs are dominated by C>T mutations at YpC sites (where Y designates C or T).

b, Relative mutation density for mutations from purines and pyrimidines in genic regions and neighboring intergenic regions of WT and XP-C cSCC sampels (SEMs are indicated).

c, TRB strength depends on the level of gene expression and is most pronounced in highly expressed genes (SEMs are indicated) specifically in XP-C cSCC samples.

d, Replication timing and intensity of mutagenesis on the transcribed and untranscribed DNA strands as well in intergenic regions of WT and XP-C cSCC samples (SEMs are indicated). The untranscribed strand for pyrimidines (or transcribed for purines, red) behaves similar to intergenic regions genome-wide due to the absence of GG-NER in XP-C tumors while this effect is weaker in WT samples due to the different mutagenesis process and functional GG-NER.

Supplementary Figure 7

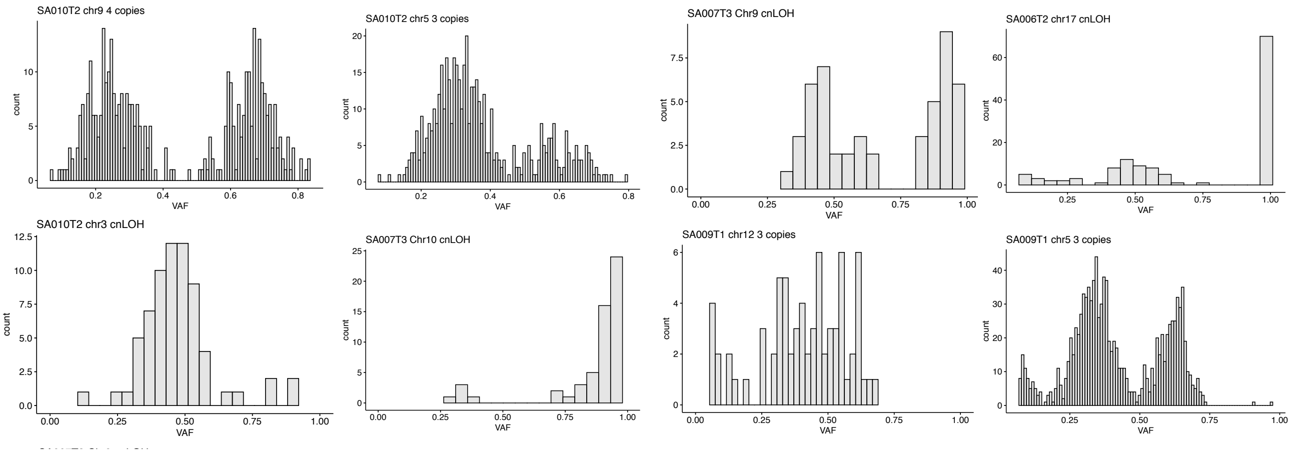

Supplementary Figure 7. Variant allele frequency distribution in SCNA regions of XP-C tumors.

Supplementary Table 1. Information about samples used in the study.

Sample	Geographic familial origin	Homozygous XPC gene mutation (1)	Diagnosis (2)	Somatic chromosomal abnormalities	Somatic putative driver mutations	Treatment before biopsy	Sequencing Machine	Mean coverage Tumor/Normal	Tumor purity (FACETS)	Tumor ploidy (FACETS)	VCF filters	Material	SBS	ID	DBS	Tumor sample
**SA009T1**	Europe	delTG	RAEB-2, AML* with MDS-related changes	Complex karyotype with del(5q), del(7q), del(20q) and subclonal del(4q)	TP53:p.S215R, p.G154V; TET2:p.C1193Y	4 doses of Azacitidine	BGISEQ-500	94/32	0.94	1.86	at least 1 read on each strand, minimial VAF=0.05	CD34	36745	3147	235	CD34+; BM
**SA002T2**	North Africa	delTG	Uterine Rhabdomyosarcoma*	Complex karyotype	N/A	no	BGISEQ-500	28/15	0.46	3.62	at least 1 read on each strand, minimial VAF=0.4	FFPE	5692	280	43	FFPE (>90% tumor cells)
**SA006T2**	North Africa	delTG	AML	Complex karyotype with UPD(17p/TP53), del(4q), del(5q), del(7q), del(13) and dup(21q)	TP53:p.V272M; RAD21:c.937+1G>T (splice)	no	BGISEQ-500	40/36	0.79	1.62	at least 1 read on each strand, minimial VAF=0.05	CD34	33230	2322	198	CD34+; BM
**SA008T6**	North Africa	delTG	RAEB-1, RAEB-2, AML-6*	Complex karyotype with del(5q)	TP53:p.E346X, c.672+1G>T (splice); CSF3R:p.P467S, c.1959-1G>A, c.1576+1G>C, c.1071+1G>C	CETUXIMAB for few monthts for skin cancers, 20 doses of Azacitidine	Illumina Hiseq 2500	65/34	0.89	1.9	at least 1 read on each strand, minimial VAF=0.05	CD34	37783	2377	245	CD34+; BM
**SA012T2**	North Africa	delTG	AML-6	Complex karyotype with del(5q)	N/A	no	BGISEQ-500	41/34	n/a (no SCNAs)	2	at least 1 read on each strand, minimial VAF=0.05	CD14	33821	2575	160	CD14+; BM
**SA007T3**	East Africa	IVS12	Breast sarcoma*	cnLOH(9q,10q)	CDKN2A homozygous deletetion	no	BGISEQ-500	43/21	0.85	1.97	at least 3 reads on each strand, minimial VAF=0.3	FFPE	4787	451	34	FFPE (>90% tumor cells)
**SA010T2**	North Africa	delTG	AML-6	del(5q), monosomy 7, del(9q), del(20q)	TP53:p.T284P	no	BGISEQ-500	23/40	0.71	1.85	at least 1 read on each strand, minimial VAF=0.05	CD34	17685	1722	99	CD34+; BM
**SA011T2**	North Africa	delTG	T-ALL, RAEB-1, AML*	T-ALL: trisomy 20; MDS: del(5q), del(7q) ; AML: complex karyotype with additional abnormalities	T-ALL:PHF6 E150*, BCOR G1056fs; MDS: TP53:p.R280X	1 year of Azacitidine	BGISEQ-500	26/32	0.75	1.83	at least 1 read on each strand, minimial VAF=0.05	CD34	17274	1464	98	CD34+; BM

(1) delTG refers to c.1643_1644 delTG; p.Val548AlafsX572, IVS12 refers to the splice site mutation NM_004628:exon13:c.2251-1G>C
(2) RAEB - Refractory Anemia with Excess Blasts, AML - Acute Myeloid Leukemia, MDS - Myelodysplastic Syndrome, T-ALL - T-cell Acute Lymphoblastic Leukemia.

*Tumor samples used for genomic sequencing.

Supplementary Table 2. Number of mutations occurred before and after SCNAs.

Sample	events	VAF_cuttoff	type_of_event	Chromosome	Total mutations in segment	Mutations before SCNA per haploid DNA copy	Mutations after SCNA per haploid DNA copy	Difference	log2 difference
SA009T1	2	0.5	3 copies	5	1070	374	107.3	3.485555	1.80138819
SA009T1	2	0.5	3 copies	12	71	24	7.6	3.157895	1.65896308
SA007T3	2	0.75	cnLOH	9	49	23	13	1.769231	0.82312224
SA007T3	2	0.75	cnLOH	10	56	51	2.5	20.4	4.35049725
SA006T2	1	0.8	cnLOH	17	130	70	30	2.333333	1.22239242
SA010T2	1	0.75	cnLOH	3	68	4	32	0.125	-3
SA010T2	1	0.45	3 copies	5	479	118	81	1.45679	0.54279305
SA010T2	1	0.45	4 copies	9	384	178	7	25.42857	4.66837851
