# Supplementary material for: XPC deficiency increases risk of hematologic malignancies through mutator phenotype and characteristic mutational signature
Source: Nat Commun. 2020 Nov 17;11:5834. doi: 10.1038/s41467-020-19633-9 (PMC7672101; doi:10.1038/s41467-020-19633-9)
Supplement: Supplementary file 3 — Reporting Summary [file 41467_2020_19633_MOESM3_ESM.pdf]

nature research

Corresponding author(s): Sergey Nikolaev
Last updated by author(s): Sep 30, 2020

Reporting Summary

Nature Research wishes to improve the reproducibility of the work that we publish. This form provides structure for consistency and transparency in reporting. For further information on Nature Research policies, see our Editorial Policies and the Editorial Policy Checklist.

Statistics

For all statistical analyses, confirm that the following items are present in the figure legend, table legend, main text, or Methods section.

n/a	Confirmed	
☐	☒	The exact sample size (n) for each experimental group/condition, given as a discrete number and unit of measurement
☒	☐	A statement on whether measurements were taken from distinct samples or whether the same sample was measured repeatedly
☐	☒	The statistical test(s) used AND whether they are one- or two-sided<br>*Only common tests should be described solely by name; describe more complex techniques in the Methods section.*
☒	☐	A description of all covariates tested
☐	☒	A description of any assumptions or corrections, such as tests of normality and adjustment for multiple comparisons
☐	☒	A full description of the statistical parameters including central tendency (e.g. means) or other basic estimates (e.g. regression coefficient) AND variation (e.g. standard deviation) or associated estimates of uncertainty (e.g. confidence intervals)
☐	☒	For null hypothesis testing, the test statistic (e.g. F, t, r) with confidence intervals, effect sizes, degrees of freedom and P value noted<br>*Give P values as exact values whenever suitable.*
☒	☐	For Bayesian analysis, information on the choice of priors and Markov chain Monte Carlo settings
☒	☐	For hierarchical and complex designs, identification of the appropriate level for tests and full reporting of outcomes
☒	☐	Estimates of effect sizes (e.g. Cohen's d, Pearson's r), indicating how they were calculated

Our web collection on statistics for biologists contains articles on many of the points above.

Software and code

Policy information about availability of computer code

Data collection	No software was used for data collection
Data analysis	QC of the reads: FASTQC (Andrews, 2015) (v0.11.7); Read mapping: BWA-MEM (v0.7.12) software (Li and Durbin, 2009) ; BAM files preparation: samtools (Li et al., 2009) (v1.9); BAM QC: mosdepth (Pedersen and Quinlan, 2018) (v0.2.5) and multiqc (Ewels et al., 2016) (v1.5); variant calling: GATK mutect2 (Depristo et al., 2011) (v4.0.10.1); annotation of the variants: oncotator (Ramos et al., 2015) (v1.9.9.0), mutational signature analysis: MutationalPatterns software (Blokzijl et al., 2018) (v.1.11.0), SigProfilerMatrixGenerator v.1.0 software (Bergstrom et al., 2019), R statistical software v3.5.1; genomic coordinates: BEDOPS v2.4.37 (bedmap) software (Neph et al., 2012), BEDTools v2.29.0 (Quinlan, 2014); SCNA analysis: FACETS v0.5.14 (Shen and Seshan, 2016); pipeline assembler: snakemake v5.4.0 (Köster and Rahmann, 2012).

For manuscripts utilizing custom algorithms or software that are central to the research but not yet described in published literature, software must be made available to editors and reviewers. We strongly encourage code deposition in a community repository (e.g. GitHub). See the Nature Research guidelines for submitting code & software for further information.

Data

Policy information about availability of data

All manuscripts must include a data availability statement. This statement should provide the following information, where applicable:

- Accession codes, unique identifiers, or web links for publicly available datasets
- A list of figures that have associated raw data
- A description of any restrictions on data availability

Experimental data generated in this study have been deposited to the European Genome-phenome Archive (EGA), the accession number is EGAS00001004511 [https://ega-archive.org/studies/EGAS00001004511]. The PCAWG data referenced in the study (consensus VCF files with SNVs and INDELs) are available in a public

repository from the https://dcc.icgc.org/repositories website. Genomic dataset of cSCC used in this study is available in dbGaP database under accession code phs000830.v1.p1 [https://www.ncbi.nlm.nih.gov/projects/gap/cgi-bin/study.cgi?study_id=phs000830.v1.p1]. All the other data supporting the findings of this study are available within the article and its supplementary information files and from the corresponding author upon reasonable request. A reporting summary for this article is available as a Supplementary Information file.

Field-specific reporting

Please select the one below that is the best fit for your research. If you are not sure, read the appropriate sections before making your selection.

- [x] Life sciences
- [ ] Behavioural & social sciences
- [ ] Ecological, evolutionary & environmental sciences

For a reference copy of the document with all sections, see nature.com/documents/nr-reporting-summary-flat.pdf

Life sciences study design

All studies must disclose on these points even when the disclosure is negative.

Sample size	We used all the available unique samples from XP-C patients and tissue-matched sporadic cancers from PCAWG
Data exclusions	No data were excluded.
Replication	Only independent biological replicates were used in this study. No technical replicates were used.
Randomization	Cancer samples were aggregated into groups based on the cancer type and XPC defficiency status.
Blinding	Blinding was not relevant for this study because we worked with collection and analysis of rare samples and their comparison with published cohorts.

Reporting for specific materials, systems and methods

We require information from authors about some types of materials, experimental systems and methods used in many studies. Here, indicate whether each material, system or method listed is relevant to your study. If you are not sure if a list item applies to your research, read the appropriate section before selecting a response.

Materials & experimental systems

n/a	Involved in the study	
[x]	[ ]	Antibodies
[x]	[ ]	Eukaryotic cell lines
[x]	[ ]	Palaeontology and archaeology
[x]	[ ]	Animals and other organisms
[ ]	[x]	Human research participants
[x]	[ ]	Clinical data
[x]	[ ]	Dual use research of concern

Methods

n/a	Involved in the study	
[x]	[ ]	ChIP-seq
[x]	[ ]	Flow cytometry
[x]	[ ]	MRI-based neuroimaging

Human research participants

Policy information about studies involving human research participants

Population characteristics	Patients from the study were diagnosed with Xeroderma Pigmentosum at early age (median: 3.5 years (range 1.5-9 years); Table 1 and Supplementary Table 1). Primary fibroblasts from sun unexposed skin were used to determine the DNA repair deficiency by unscheduled DNA synthesis following UV-C irradiation as described (Sarasin et al., 1992). The XP genetic defect was characterized by complementation assay using recombinant retroviruses expressing wild type DNA repair genes (Arnaudeau-Bégard et al., 2003).
Recruitment	The study participants were patients with Xeroderma pigmentosum. These patients were treated in hospitals of France. There was no specific selection of the patients except their diagnosis.
Ethics oversight	Informed signed consents were obtained from patients and/or their parents in accordance with the Declaration of Helsinki and the French law. This study was approved by the French Agency of Biomedicine (Paris, France), by the Ethics Committee from the CPP of Universitary Bordeaux Hospital (Bordeaux, France) and by the Institutional Review Board of the University Institute of Hematology (IUH: Saint-Louis Hospital, Paris).

Note that full information on the approval of the study protocol must also be provided in the manuscript.
