# Supplementary material for: XPC deficiency increases risk of hematologic malignancies through mutator phenotype and characteristic mutational signature
Source: Nat Commun. 2020 Nov 17;11:5834. doi: 10.1038/s41467-020-19633-9 (PMC7672101; doi:10.1038/s41467-020-19633-9)
Supplement: Supplementary file 4 — Description of Additional Supplementary Files [file 41467_2020_19633_MOESM4_ESM.pdf]

Description of Additional Supplementary Files

File Name: Supplementary Data 1
Description: Matrixes of single, double substitutions and indels in different mutational classes.

File Name: Supplementary Software 1
Description: Supplementary code used to calculate probability of mutation clusterisation.
